# Carnosol and Related Substances Modulate Chemokine and Cytokine Production in Macrophages and Chondrocytes

**DOI:** 10.3390/molecules21040465

**Published:** 2016-04-08

**Authors:** Joseph Schwager, Nathalie Richard, Ann Fowler, Nicole Seifert, Daniel Raederstorff

**Affiliations:** DSM Nutritional Products, Wurmisweg 576, P. O. Box 2676, Basel 4002, Switzerland; Nathalie.Richard@dsm.com (N.R.); Ann.Fowler@dsm.com (A.F.); Nicole.Seifert@dsm.com (N.S.); Daniel.Raederstorff@dsm.com (D.R.)

**Keywords:** abietanes, carnosol, chondrocyte, osteoarthritis, murine macrophages, innate immune response, acute inflammation, chronic inflammation, NF-κB signaling pathway and nuclear translocation

## Abstract

Phenolic diterpenes present in *Rosmarinus officinalis* and *Salvia officinalis* have anti-inflammatory and chemoprotective effects. We investigated the *in vitro* effects of carnosol (CL), carnosic acid (CA), carnosic acid-12-methylether (CAME), 20-deoxocarnosol and abieta-8,11,13-triene-11,12,20-triol (ABTT) in murine macrophages (RAW264.7 cells) and human chondrocytes. The substances concentration-dependently reduced nitric oxide (NO) and prostaglandin E_2_ (PGE_2_) production in LPS-stimulated macrophages (*i.e.*, acute inflammation). They significantly blunted gene expression levels of iNOS, cytokines/interleukins (IL-1α, IL-6) and chemokines including CCL5/RANTES, CXCL10/IP-10. The substances modulated the expression of catabolic and anabolic genes in chondrosarcoma cell line SW1353 and in primary human chondrocytes that were stimulated by IL-1β (*i.e.*, chronic inflammation In SW1353, catabolic genes like MMP-13 and ADAMTS-4 that contribute to cartilage erosion were down-regulated, while expression of anabolic genes including Col2A1 and aggrecan were shifted towards pre-pathophysiological homeostasis. CL had the strongest overall effect on inflammatory mediators, as well as on macrophage and chondrocyte gene expression. Conversely, CAME mainly affected catabolic gene expression, whereas ABTT had a more selectively altered interleukin and chemokine gene exprssion. CL inhibited the IL-1β induced nuclear translocation of NF-κBp65, suggesting that it primarily regulated via the NF-κB signalling pathway. Collectively, CL had the strongest effects on inflammatory mediators and chondrocyte gene expression. The data show that the phenolic diterpenes altered activity pattern of genes that regulate acute and chronic inflammatory processes. Since the substances affected catabolic and anabolic gene expression in cartilage cells *in vitro*, they may beneficially act on the aetiology of osteoarthritis.

## 1. Introduction

**Figure 1:**
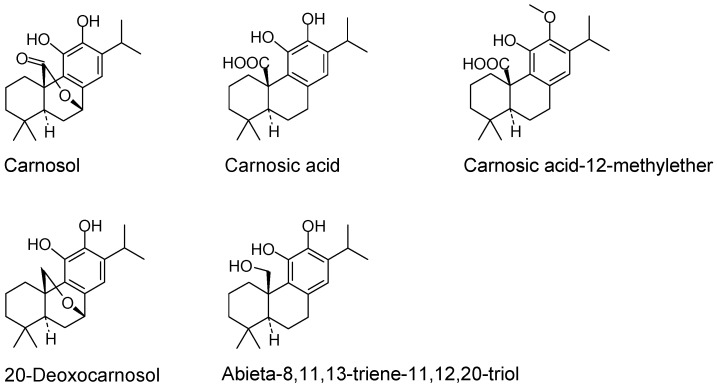
Structures of the abietane diterpenes used in this study.

Chronic inflammatory processes are implicated in the etiology of many diseases, including arthritis, atherosclerosis, diabetes and neurodegenerative disorders. Natural substances including phytochemicals can modulate a variety of these processes by interacting with receptors and signaling pathways, modulating enzyme activities or regulating gene expression via transcription factors. These phytochemicals may thereby modulate cell proliferation and differentiation and potentially influence disease development. Carnosol (CL) and structurally related diterpenes are major secondary plant metabolites in Lamiaceae spp. such as *Salvia officinalis*, *Rosmarinus officinalis* and *Origanum vulgare* that exhibit numerous biological features (Figure 1). Anti-oxidant properties of CL, carnosic acid (CA) or carnosic acid-12 methylether (CAME) have been demonstrated by different physicochemical methods [1,2,3,4,5,6]. CL and CA act on cell proliferation and may be therefore chemopreventive and anti-tumorigenic [4,7,8,9,10,11,12]. Anti-inflammatory effects were substantiated in various cellular systems [13,14,15,16,17,18,19] and initially shown by the inhibition of nitric oxide (NO) production [3,4]. At the molecular level, CL activates genes through anti-oxidant response elements [20,21], regulating gene expression along signaling pathways [4,22] and binding of transcription factors to promoter elements [23].

We investigated anti-inflammatory effects of CL and structurally related abietane diterpenes in macrophages and chondrocytes and thus significantly extend previous studies [3,4,24]. Most of them potently inhibited LPS-induced NO production and COX-2 dependent prostaglandin E_2_ (PGE_2_) production. Here, we demonstrate that abietane diterpenes modulated the expression level of cytokine and chemokine genes in macrophages (reflecting both acute and chronic inflammation) and chondrocytes, where pathophysiological conditions related to chronic inflammation and osteoarthritis (OA) were induced [25,26,27,28,29]. Therefore these molecules could offer novel perspectives in the treatment of diseases where inflammation and cartilage destruction are predominant feature like in OA [30].

## 2. Results

### 2.1. Anti-Inflammatory Effects Measured in Macrophages

Murine macrophage RAW264.7 cells were stimulated with lipopolysaccharide (LPS) to induce inflammatory mediators including NO and PGE_2_ and the inhibitory effects of CL and related abietane diterpenes were determined. CL potently reduced the production of both NO and PGE_2_, with IC_50_ of 5.0 ± 2.8 and 9.4 ± 2.1 μM, respectively (Table 1) (see also [4]). Related compounds such as CA, carnosic acid 12-methyl ether (CAME), 20-deoxocarnosol and abieta-8,11,13-triene-11,12,20 triol (ABTT) had comparable effects on NO production (except for 20-deoxocarnosol and ABTT), yet they inhibited PGE_2_ production less efficiently. It should be noted that the substances outperformed NG-nitro-l-arginine methyl ester (l-NAME), which is an inhibitor of iNOS-2 and thus blocks the production of NO in macrophages. None of the tested substances significantly affected cell viability at any concentrations as determined by the LDH release assay (data not shown). We also observed that CL, 20-deoxocarnosol and ABTT inhibited the *in vitro* enzyme activity of cyclooxygenase (COX)-1 and/or COX-2 with IC_50_ > 200 μM and thus at physiologically irrelevant concentrations (A.F. data not shown).

### 2.2. Abietane Diterpenes Modulate the Expression of Cytokine and Chemokine Genes in Macrophages

**Figure 2:**
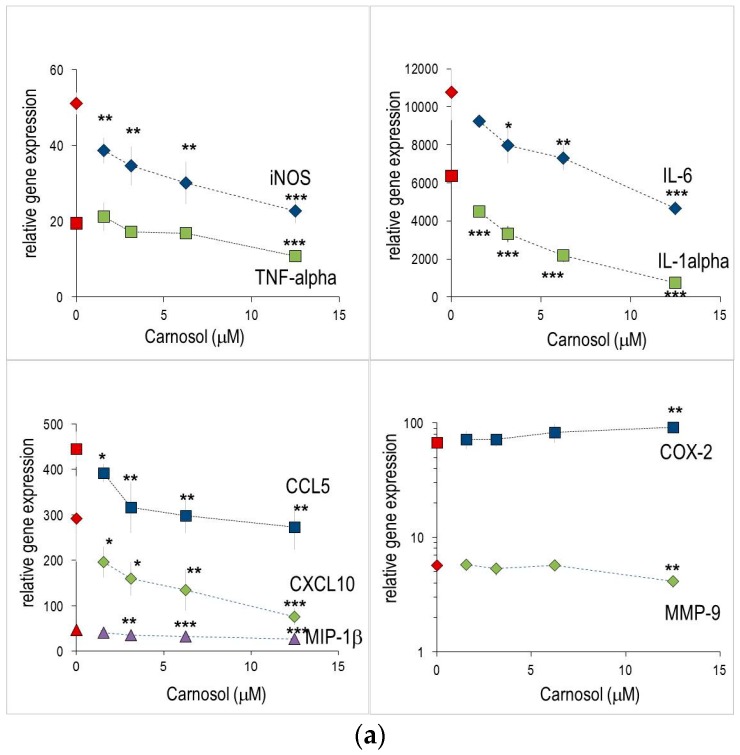

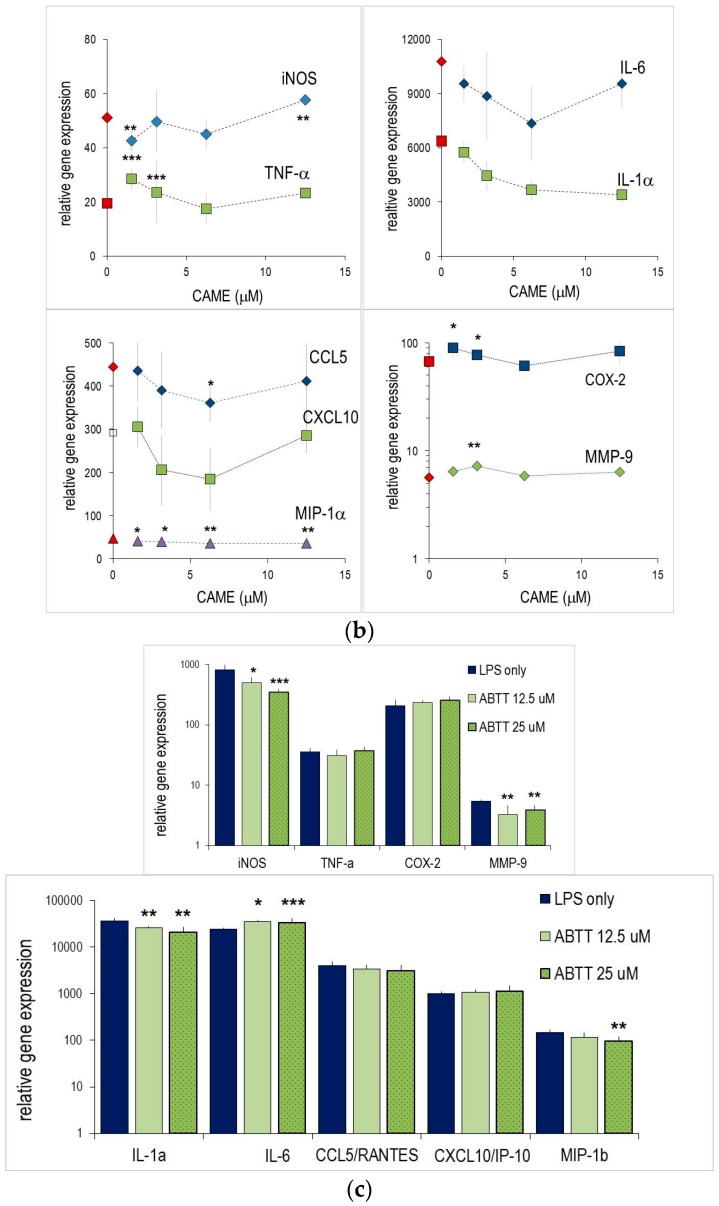
Abietane diterpenes down-regulated gene expression in RAW264.7 cells. Cells were stimulated with LPS in the presence of indicated amounts of substances for 4 h and the levels of mRNA determined by quantitative RT-PCR. “Relative gene expression”, which indicates gene expression levels in stimulated relative to unstimulated cells, was calculated as described in Materials and Methods. Asterisks indicate statistical significant differences compared to LPS-stimulated cells (* *p* < 0.05, ** *p* < 0.01, *** *p* < 0.001; *n* = 3). Effect of carnosol (**a**); carnosic acid 12-methylether (CAME) (**b**) and abieta-8,11,13-triene-11,12,20 triol (ABTT) (**c**).

We investigated whether CL, CAME and ABTT affected the production of inflammatory mediators at the transcriptional level and thus changing the respective gene expression. To this aim, mRNA levels were measured in macrophages, which were treated for 4 h. All substances were tested at a range that encompassed the IC_50_ values established for NO or PGE_2_ production. The substances *per se* did not influence basal mRNA levels in unstimulated cells. LPS treatment significantly increased the expression of inflammatory genes such as COX-2, inducible nitric oxide synthase (iNOS), TNF-α, IL-1α, CCL4/MIP-1β, CCL5/RANTES and CXCL10/IP-10 (Supplementary Material Table S1). CL concentration-dependently diminished the expression levels of several genes (Figure 2a). For instance, iNOS, IL-1α, IL-6 or CXCL10/IP-10 were significantly reduced even at the lowest CL concentration tested (1.56 μM); at higher concentrations mRNA levels were reduced by up to 90% (e.g., IL-6). Conversely, CL did not alter expression level of e.g., COX-2, CCL4/MIP-1β or MMP-9 in LPS-stimulated murine macrophages. While CAME had small effects on IL-6 and IL-1α (Figure 2b), it failed to modulate expression of iNOS, TNF-α and CCL5/RANTES and CXCL10/IP-10 genes. Consistent with the observed effects on NO production, ABTT also impaired iNOS mRNA levels, but had only marginal effects on other inflammatory gene, and therefore displayed similar activities as CAME (Figure 2c). It should be noted, that CAME and ABTT increased expression of IL-6 or MMP-9. CL and CAME (at 12.5 μM) induced COX2 expression and iNOS expression, respectively.

### 2.3. Carnosol and Related Diterpenes Impair Expression of Chemokine and Catabolic Genes in Chondrosarcoma Cells and Primary Chondrocytes

**Figure 3:**
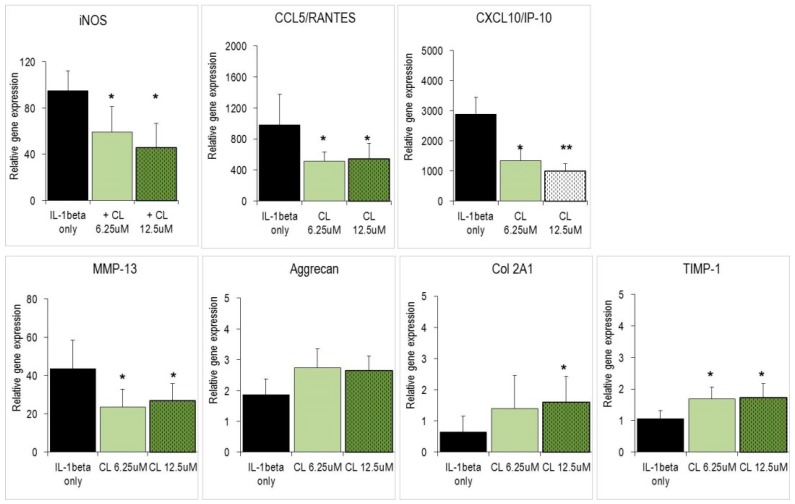
Effect of substances on IL-1β activated chondrosarcoma SW1353 cells. Cells were stimulated with IL-1β for 4 h in the presence of indicated substances. Gene expression was determined by quantitative RT-PCR as described in Figure 2. Asterisks indicate statistical significant differences compared to IL-1β stimulated cells (* *p* < 0.05, ** *p* < 0.01, *n* = 4).

Since CL is a potent anti-inflammatory substance, it might induce changes in biological systems where chronic inflammation impairs cell and tissue homeostasis. Thus, we explored effects of CL on the chondrosarcoma SW1353 cells, which are a substitute for primary human chondrocytes and an *in vitro* surrogate for osteoarthritic tissue. SW1353 cells expressed a comparable although not identical set of genes as primary chondrocytes when they were activated with IL-1β [25,28,29]. Specifically, activated SW1353 cells strongly augmented gene expression levels of catabolic genes, *i.e.*, matrix metalloproteinase (MMP)-3 and MMP-13, which are critically involved in erosion of the extracellular matrix (ECM) (Supplementary Material Table S2). Whereas IL-1β treatment only slightly affected expression levels of anabolic genes (*i.e.*, aggrecan, collagen), it triggered a strong increase of chemokine gene expression, with CCL5/RANTES and CXCL10/IP-10 being the most responsive members (Supplementary Material Table S2). CL significantly diminished the expression levels of these genes (Figure 3). In contrast, expression of anabolic genes including aggrecan and Col2A1 as well as the anti-catabolic TIMP-1 was increased by CL.

**Figure 4:**
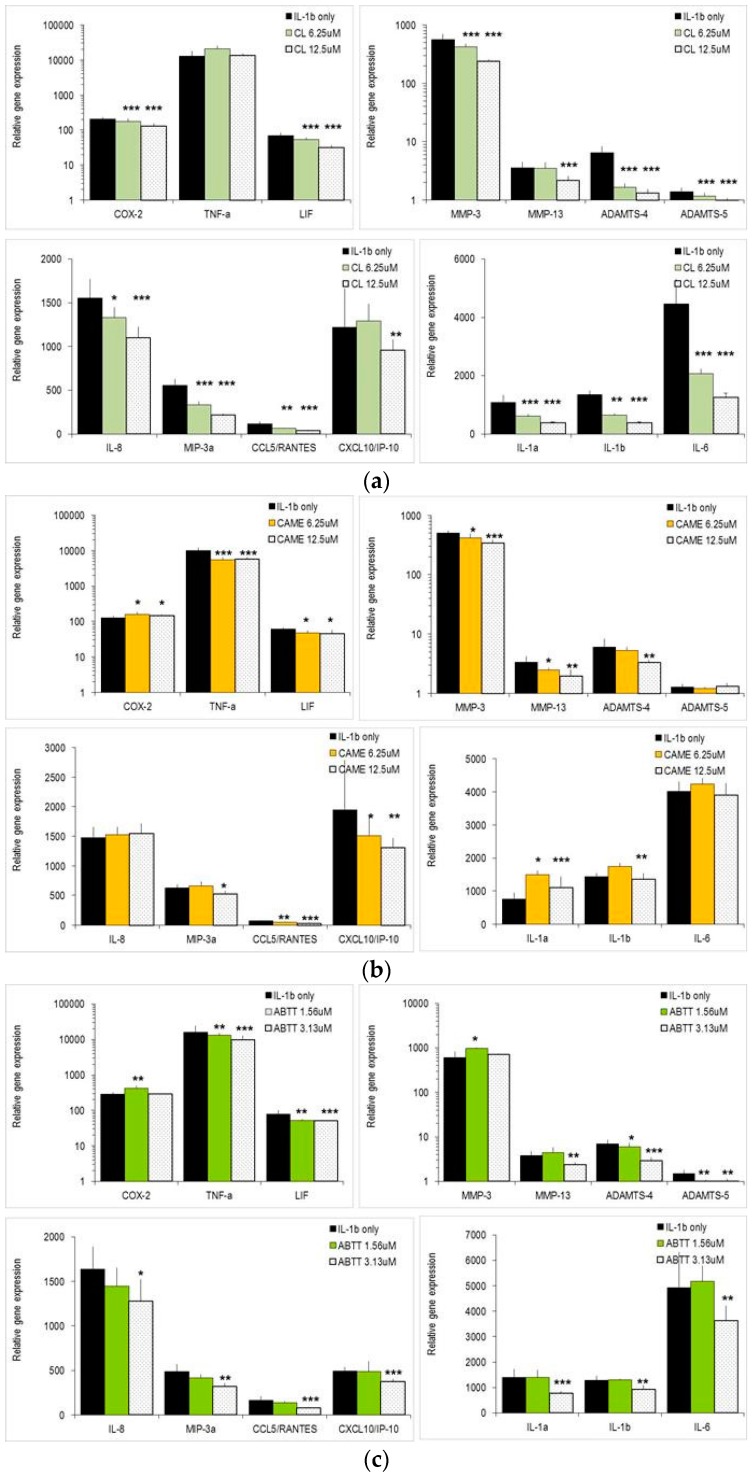
Effect of substances on IL-1β activated NHAC-kn. Cells were stimulated with IL-1β for 4 h in the presence of indicated substances. Gene expression was determined by quantitative RT-PCR as described in Figure 2. Asterisks indicate statistical significant differences compared to IL-1β stimulated cells (* *p* < 0.05, ** *p* < 0.01, *** *p* < 0.001; *n* = 4). (**a**): Effect of carnosol (CL); (**b**): effect of carnosic acid 12-methylether (CAME); (**c**): effect of abieta-8,11,13-triene-11,12,20 triol (ABTT). Note log scale on the y axis in some panels.

Next, we studied the effects of CL on normal human articular chondrocytes from knee (NHAC-kn), which were activated with IL-1β. The IL-1β induced changes in NHAC-kn gene expression levels were comparable to those observed in SW1353 cells [28,29]. IL-1β significantly up-regulated catabolic genes (MMP-3, MMP-13, ADAMTS-4), interleukins (IL-1α, IL-1β, IL-6), chemokines (CXCL8/IL-8, CCL20/MIP-3α, CCL5/RANTES, CXCL10/IP-10) but also COX-2, TNF-α and LIF (Figure 4). CL significantly impaired the expression of three chemokine genes (CXCL8/IL-8, CCL20/MIP-3α, CCL5/RANTES) (Figure 4a). Similarly, IL-1α, IL-1β and IL-6 were concentration-dependently down-regulated by CL. It had no significant effect on TNF-α expression and only slightly down-regulated COX-2 and LIF. With regard to catabolic genes, MMP-3, MMP-13, ADAMTS-4 and ADAMTS-5 were robustly reduced by CL at 6.25 μM and 12.5 μM. In contrast, CAME impaired chondrocyte gene expression in a more restricted way (Figure 4b): it rather increased the expression levels of IL-1α, IL-1β and did not change IL-6 or chemokine genes (CXCL8/IL-8, CCL20/MIP-3α, CCL5/RANTES). However, it affected CXCL10/IP-10 and the catabolic genes MMP-3, MMP-13 and ADAMTS-4. ABTT exerted still another activity pattern (Figure 4c): three of four chemokine genes were concentration-dependently reduced by ABTT, while interleukins including IL-1α, IL-1β, and IL-6 were only affected by high concentrations of ABTT (25 μM). Unlike CL or CAME, ABTT had no major impact on mRNA levels of most catabolic genes. Collectively, these findings show that CL reduced the expression of genes involved in erosion of ECM and increased the expression levels of anabolic genes (in SW1353 chondrosarcoma cells). Furthermore it modulated gene expression of different chemokines and pro-inflammatory interleukins, whereas CAME and ABTT affected gene expression of cartilage-degrading enzymes and chemokines, respectively.

### 2.4. Nuclear Translocation of NF-κB

**Figure 5:**
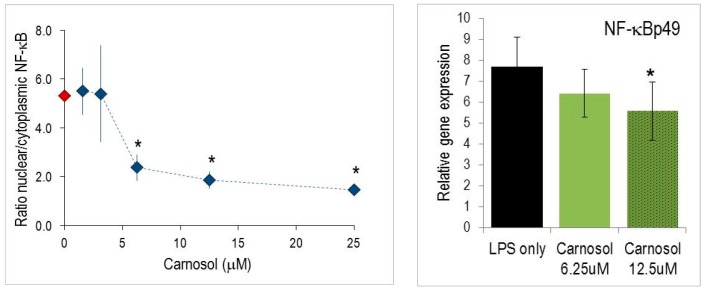
Carnosol altered the NF-kB signaling pathway. Left panel: Nuclear translocation of NF-κB. Chondrocytes (NHAC-kn) were pre-incubated with carnosol for 1 h and stimulated with IL-1β for 20 min. The relative nuclear immunofluorescence is indicated on the y-axis (see Materials and Methods for details). Asterisks indicate statistically significant differences between the different treatments (* *p*< 0.05). Right panel: Carnosol reduces expression of transcription factors of the NF-κB pathway. Cells were stimulated for 4 h and gene expression levels determined by RT-PCR (* *p* < 0.05).

Gene activation is dependent on early events in the NF-κB signaling pathway. In order to further investigate the mode of action of CL in chondrocytes, the nuclear translocation of NF-kB was measured by Arrayscan™ cytometry (see Materials and Methods) [31]. IL-1β activated cells responded within 20 min by a substantial shift of NF-κBp65 to the cell nucleus, which was reflected by the appropriate shift in the ratio of nuclear/cytoplasmic fluorescence (Figure 5). The translocation of NF-κBp65 in the nucleus was significantly reduced by increasing concentrations of CL.

## 3. Discussion

The range of biological properties of CL, CA, CAME and ABTT described in the present study underscores their multiple effects on acute and chronic inflammation. These substances, which form a significant part of the dry plant mass in rosemary, oregano, and sage [4,5,32,33], influence both the production of inflammatory metabolites and the expression of inflammatory genes. It should be emphasized that the biological activities of the substances were analyzed in two diverse cellular models, *i.e.*, macrophages and chondrocytes, the latter being critically involved in OA. More specifically, the study shows for the first time the effects of abietane diterpenes on activated chondrocytes and point at their possible use in OA. As convincingly shown in a recent study [30], carnosol potently inhibited pro-inflammatory cytokines and chemokines and reduced the production of mediators involved in ECM breakdown.

NO and PGE_2_ have important functions in acute and chronic inflammatory processes. In general, their inhibition is considered as a means of disease modification and pain relief, respectively. The IC_50_ values established for CL in this study are in good agreement with the reported data on NO production in macrophages [3,4,16] and extend the findings to CA, CAME, ABTT, 20-deoxocarnosol, whose effects were comparable to those of CL (Table 1). Part of this effect might be due to the NO scavenging activity of the substances when they are added to the cellular systems [2,4]. Moreover, most of the substances markedly reduced the production of COX-2 dependent PGE_2_ in murine macrophages. Similar effects had been reported before in epithelial cells [23] but not in macrophages. With regard to eicosanoid production, CL may interact with the thromboxane (TX) A2 receptor [34] without impairing the biosynthesis of TXA nor cyclooxygenase activity [35].

We have investigated the effect of substances on the inflammatory response at the transcriptional level, since CL is expected to influence gene expression regulated by NF-κB [4] or AP-1 [23]. Nuclear translocation of NF-κBp65 was markedly impeded by CL (Figure 5 and [4,22]. As a consequence, it concentration-dependently reduced iNOS gene expression and some other (but not all) inflammatory genes that are under the control of the NF-κB signaling pathway. On the other side, COX-2 mRNA levels in murine macrophages were not impaired (Figure 2). Presumably, prostaglandin E_2_ synthase is targeted by the substances rather than COX-2 and thus accounts for reduced PGE_2_ production (Table 1). Indeed, unchanged or even increased COX-2 expression might be beneficial since COX-2 activity is required during the resolution of the *acute* inflammatory response (for a review see [36]). It should be emphasized that in *chronic* inflammation, COX-2 inhibitors are a paramount pharmacological target of widely proven efficacy for pain relief. CL had pleiotropic effects on gene expression, since pro-inflammatory cytokine expression levels (*i.e.*, IL-1α and IL-6) were also altered. During the inflammatory response to LPS, macrophages also up-regulated numerous members of other gene families, including chemokines. CXCL10/IP-10, CCL5/RANTES and CCL4/MIP-1β were among the most responsive (Supplementary Material Table S1). CL significantly reduced mRNA levels of CXCL10/IP-10 and CCL5/RANTES, whereas CCL4/MIP-1β expression was refractory to CL. Thus, the observed modulation of gene expression levels does not correlate with the presence of one common regulatory element (such as NF-κB binding element), suggesting complex interactions of CL and different transcription factors (NF-κB, AP-1) and signaling pathways (MAPK) as described for epithelial cells [23]. Also, via its genuine anti-oxidant properties, CL influences cellular redox potential and thus the glutathione and superoxide dismutase activities. This represents an additional means to control expression levels of genes involved in the inflammatory response [15,18].

Among the tested substances CL was the most efficacious, both in its effect on the production of inflammatory metabolites and the modulation of gene expression. Presumably, the five tested diterpenes (Figure 1 and Table 1) differ in their anti-oxidant properties [6,18]. In line with this, CL modulated the cellular redox potential and thus the glutathione and superoxide dismutase activities [18,20]. This represents an additional means to control expression levels of genes involved in the inflammatory response. It should be noted that the mode of action of the diterpenes is idiosyncratic, since the extent and changes of inflammatory gene expression is only partially overlapping (see Figure 2). Plausibly, the features of each substance (Figure 1) shape substance-specific interactions with factors that control gene expression.

The *in vivo* relevance of these data has to be examined in the light of achievable plasma concentrations and *in extenso* systemic levels of the substances. In a murine colonic carcinogenesis model, the inclusion of 0.1% CL in the diet reduced adenoma formation [11] and a comparable dietary intake significantly changed liver glutathione-*S*-transferase and NAD(*P*)*H*-quinone reductase [37]. In mice fed CL, steady-state plasma CL concentrations reached 4.2 μM (H. Mohajeri, personal communication); this is close to the *in vitro* IC_50_ for NO (Table 1). Intra-gastrically applied CA attained plasma concentrations of 42.5 mg/L CA (*i.e.*, 130 μM) within ~2 h [36] in rats. Hence, CL and CA plasma concentrations were well in the range of IC_50_ values that are needed to significantly modulate molecular and cellular parameters.

Finally, IL-1β is considered the key molecule that triggers osteoarthritis (OA), while both NO and PGE_2_ play a role in the OA development [27,38,39,40]. Increased production of NO in OA tissue contributes to a slowly progressing inflammation [40,41]. Here we show for the first time, that the phenolic diterpene CL concentration-dependently reduced IL-1β expression levels in macrophages and chondrocytes. This suggests that these substances might prevent or delay disease initiation and progression. More importantly, CL significantly modulated the expression of different genes that are pivotal in OA etiology: Catabolic genes (MMPs, ADAMTS) encode for enzymes that erode the extracellular matrix (ECM) in articulate tissue. As shown in Figure 3 and Figure 4, gene expression of cartilage-degrading enzymes (MMP-3, MMP-13, ADAMTS-4) was repressed by various abietane diterpenes, while anabolic genes like Col2A1 and aggrecan were up-regulated. It should be noted that other MMPs like MMP-9 and MMP-2 were not affected by CL (Figure 2, and data not shown). Hence, CL has an activity pattern that fulfils most of the requirements for a chondro-protective and cartilage-regenerating substance. Also, chondrocytes responded to IL-1β stimulation by expressing chemokine genes that are crucial for the recruitment of different cell populations to the sites of inflammation [28]. A CL-dependent reduction of CCL5/RANTES and CXCL10/IP-10 is expected to impair the recruitment of neutrophils and activated T lymphocytes, respectively, to sites of inflammation. The same cell populations are targeted by two other chemokines, CXCL8/IL-8 and CCL20/MIP-3α, which are also down-regulated in chondrocytes by the tested substances. Via these subtle changes of various chemokines the cell migration in response to inflammatory stimuli may be reduced. As a corollary of the multiple biological effects, dietary supplementation with abietane diterpenes is expected to significantly modify OA disease development and severity.

## 4. Materials and Methods

### 4.1. Phytochemicals and Reagents

Carnosol (CL), carnosic acid (CA), carnosic acid 12-methyl ester (CAME), 20-deoxocarnosol and abieta-8,11,13-triene-11,12,20 triol (ABTT) (Figure 1) were from Cayman Chemicals (Ann Arbor, MI, USA) or Fluorochem Ltd. (Glossop, Hadfield, UK) and also isolated from *Salvia officinalis* or *Rosmarinus* *officinalis* applying solvent extraction and reverse-phased chromatography. Structure elucidation of defined peaks of the resulting chromatogram was done by ^1^H-NMR and 2D-NMR. Compounds were dissolved in DMSO and added to the culture medium concomitantly with the stimulus. Final DMSO concentration in culture medium was 0.5%. *E. coli* LPS (serotype 055:B5) and fetal bovine serum (FBS) were from Sigma (St. Louis, MO, USA). DMEM and non-essential amino acids (NEAA) were from Invitrogen (Carlsbad, CA, USA). *N*(*G*)-nitro-l-arginine methyl ester (l-NAME) was from Sigma. Human IL-1β was from PeproTech EC (London, UK).

### 4.2. Cell Culture

RAW264.7 macrophage cells were from ATCC (Manassas, VA, USA) and cultured in DMEM supplemented with 50 U/mL penicillin, 50 μg/mL streptomycin, 0.1 mM NEAA (DMEM-C) and 10% FBS. Cells were seeded into 12-well or 96-well plates at 1 and 0.05 × 10^6^ cells per well, respectively, and used after 2 days of pre-culture. Cells were starved for 18 h in DMEM-C containing 0.25% FBS before the start of treatment and stimulated with lipopolysaccharide (LPS) (1 μg/mL) for 4–24 h in phenol red-free DMEM-C containing 0.25% FBS. Test substances were added concomitantly with the stimulus.

SW1353 chondrosarcoma cells were from ATCC and cultured in DMEM-C containing 10% FBS [29]. Cells were seeded into 6-well plates at 0.5 × 10^6^ cells per well. Sub-confluent cell monolayers were washed and incubated overnight in DMEM-C containing 0.25% FBS and 0.2% lactalbumin hydrolysate (Bacto™ LC, Becton Dickinson, Franklin Lakes, NJ, USA). Cells were activated with 10 ng/mL IL-1β in phenol-red free DMEM-C supplemented with 0.25% FBS and 0.2% lactalbumin hydrolysate without or with test compounds for 4–24 h. Normal human articular chondrocytes obtained from knee (NHAC-kn) were from Lonza (Basel, Switzerland) and cultured in chondrocyte growth medium (Lonza). Cells were seeded into 6-well plates at 0.5 × 10^6^ cells per well and activated with IL-1β (10 ng/ml) for 4–24 h. Cells were lysed in RLT buffer (Qiagen, Hilden, Germany) after 2–4 h of culture and total RNA was extracted. Culture supernatants were harvested after 24 h of culture and stored at −80°C.

### 4.3. RNA Isolation, cDNA Synthesis and RT-PCR

Total RNA was isolated using the RNeasy Mini Kits (Qiagen) as described [42]. RNA quality and quantity was assessed by Nanodrop^®^ ND-1000 and evaluated by the ND-1000 3.2.1 software (Witec AG, Littau, Switzerland). Total RNA was transcribed into first strand cDNA using the Superscript™ First-Strand Synthesis System for RT-PCR from Invitrogen. Real-time PCR analysis was performed with the ABI PRISM^®^ 7700 Sequence Detection System or the ABI 7900HT Fast Real-Time PCR System (Applied Biosystems (ABI), Foster City, CA, USA). Primers and probes were designed with the Primer Express™ software purchased from ABI. PCR was done using the Taqman^®^ universal PCR Master Mix (ABI). 18S rRNA primers and probes were used as internal standards. Relative gene expression quantification was done by subtracting threshold cycles (C_T_) for ribosomal RNA from the C_T_ of the targeted gene (ΔC_T_). Relative mRNA levels were then calculated as 2 ^−ΔΔCT^ (fold change), where ΔΔC_T_ refers to the ΔC_T_ of unstimulated minus treated cells. The indicated values were obtained from at least three independent series of experiments, where each treatment was done in duplicates with each being analyzed twice in RT-PCR.

### 4.4. Measurement of Nitric Oxide and PGE_2_ Determination

The concentration of NO in culture supernatants was measured using the Griess Reaction [43]. Secreted PGE_2_ was determined by Enzyme Immuno Assay (EIA) (Cayman Chemicals).

### 4.5. NF-κB Translocation Experiments in Chondrocytes

NHAC-kn chondrocytes were grown in 96-well plates to sub-confluence. They were pre-incubated with various concentrations of CL for 1 h. NHAC-kn were activated with IL-1β for 20 min. Thereafter, cells were washed, fixed and permeabilized as described [31]. Immunostaining for NF-κBp65 was performed using the NF-κB Activation HitKit™ (Cellomics™ Inc., Pittsburgh, PA, USA). Nuclei were counter-stained with Hoechst dye. Immunofluorescence was measured by quantitative cytometric technique, Arrayscan™, with the Cellomics instrumentation (Cellomics™ Inc.) using the instruments settings as detailed before [27] and expressed as Mean_CircRingAvgIntenRatioCh2 (for detail see: Cellomics HCS application guide at http://www.cellomics.com). All treatments were done in triplicates.

### 4.6. Statistical Analysis

Data were obtained from at least three independent series of experiments and presented as means +/− standard deviation. *p* values <0.05 (calculated by Student’s t test or one way ANOVA) were considered statistically significant.

## 5. Conclusions

Carnosol and related abietanes from *Salvia* and *Rosmarinus* potently modulated cytokines, chemokines and genes implicated in cartilage erosion. Hence they beneficially influenced acute and chronic inflammatory processes in macrophages and chondrocytes.

## Figures and Tables

**Table 1:** IC_50_ values for abietane diterpene in NO and PGE_2_ production.

	Carnosol (CL)	Carnosic Acid 12-Methyl Ether (CAME)	20-Deoxy-Carnosol	Carnosic Acid (CA)	Abieta-8,11,13-Triene-11,12,20 Triol (ABTT)	l-NAME ^1^
NO	5.0 ± 2.8	3.8 ± 0.6	11.9 ± 0.6	6.9 ± 2.2	12.5 ± 4.8	150 ± 34
PGE_2_	9.4 ± 2.1	44.4 ± 6.1	18.8 ± 6.4	11.4 ± 0.9	7.8 ± 4.6	>1000

^1^ NG-nitro-l-arginine methyl ester. RAW 264.7 cells were stimulated with LPS for 24 h in the presence of varying concentrations of compounds (0.16–50 μM). NO and PGE_2_ were measured in culture supernatants and the IC_50_ values were calculated. Values are means ± standard deviation of at least three independent experimental series. Numbers indicate means ± SD (in μM) of IC_50_ values for NO and PGE_2_ (*n* = 5).

## Supplementary Materials

Supplementary materials can be accessed at: http://www.mdpi.com/1420-3049/21/4/465/s1.

## Abbreviations

The following abbreviations are used in this manuscript:

ABTT	8,11,13-Abietatriene-11,12,20-triol
CA	Carnosic acid
CL	Carnosol
CAME	Carnosic acid 12-methyl-ether
NHAC-kn	Normal human articulocytes-knee
NO	Nitric oxide
PGE_2_	Prostaglandin E_2_
