# Supplementary material for: Carnosol and Related Substances Modulate Chemokine and Cytokine Production in Macrophages and Chondrocytes
Source: Molecules. 2016 Apr 8;21(4):465. doi: 10.3390/molecules21040465 (PMC6274263; doi:10.3390/molecules21040465)
Supplement: Supplementary file 1 [file molecules-21-00465-s001.pdf]

Supplementary Materials: Carnosol and Related Substances Modulate Chemokine and Cytokine Production in Macrophages and Chondrocytes

Joseph Schwager , Nathalie Richard, Ann Fowler, Nicole Seifert and Daniel Raederstorff

Table S1. Gene expression levels in unstimulated and LPS-stimulated murine macrophages RAW264.7.

Gene	Type of Expression	Time	Unstimulated Cells	LPS Stimulated Cells	Fold Change [1]
			Δ C_T ± stdev [2]	Δ C_T ± stdev [2]	
COX-2	Intermediate	4 h	15.81 ± 0.40	9.75 ± 0.10	48
iNOS	Intermediate	4 h	16.43 ± 0.19	10.75 ± 0.08	52
TNF-α	High	4 h	13.39 ± 0.05	9.10 ±0 .07	20
IL-1α	Low	4 h	22.90 ± 0.25	10.26 ± 0.08	6650
IL-1β	Low	4 h	31.92 ± 0.23	20.68 ± 0.25	1260
IL-6	Low	4 h	29.83 ± 1.97	16.44 ±0.18	10800
IL-10	Low	4 h	26.34 ± 0.82	23.64 ± 0.36	6
PGES	Low	4 h	24.17 ± 0.26	23.11 ± 0.04	2
PGDS	Intermediate	4 h	17.12 ± 0.04	18.23 ± 0.19	0.5
MMP-9	High	4 h	14.03 ± 0.04	11.52 ± 0.12	6
CCL4/MIP-1β	High	4 h	14.42 ± 0.29	8.86 ±0 .14	190
CCL5/RANTES	Intermediate	4 h	19.30 ± 0.16	10.50 ± 0.12	450
CXCL10/IP-10	Intermediate	4 h	19.26 ± 0.11	11.07 ± 0.40	290
NF-κB1	High	4 h	13.83 ± 0.08	11.56 ± 0.10	5
NF-κB49	High	4 h	14.97 ± 0.17	12.92 ± 0.13	4
NF-κBp65	High	4 h	13.01 ± 0.04	13.12 ± 0.04	1
I-κBα	High	4 h	13.73 ± 0.21	11.57 ± 0.08	5
CD14	High	4 h	14.05 ± 0.16	12.81 ± 0.10	0.4

[1]: $2^{-\Delta\Delta CT}$; $\Delta\Delta C_T$ refers to the ΔC_T of unstimulated minus LPS-treated cells. [2]: (threshold cycles for 18S rRNA [internal standard]—threshold cycles for genes of) ± st.dev. Cells were cultured for 4 h in the absence or presence of 1 µg/mL LPS. Gene expression levels were measured by quantitative real time PCR and classified as 'high' (Δ C_T < 15), 'intermediate' ((Δ C_T 15–25) and 'low' ((Δ C_T > 25).

Table S2. Comparison of gene expression levels in unstimulated and IL-1β-stimulated SW1353 and primary chondrocytes NHAC-kn.

Gene	Time	SW1353		NHAC-kn	
		Unstimulated	IL-1b Stimulated	Unstimulated	IL-1b Stimulated
		ΔC_T ± st.dev. [1]	ΔC_T ± st.dev. [1]	ΔC_T ± st.dev. [1]	ΔC_T ± st.dev. [1]
COX-2	4 h	22.76 ± 0.87	19.30 ± 0.33	16.76 ±0.09	9.80 ± 0.18
iNOS	4 h	25.70 ± 0.64	19.13 ± 0.24	nd	nd
TNF-α	4 h	25.59 ± 1.07	14.19 ± 0.22	28.98 ± 0.63	15.70 ± 0.25
IL-1α	4 h	23.19 ± 0.48	17.97 ±0.18	26.86 ± 0.27	17.30 ± 0.32
IL-1β	4 h	nd	nd	19.57 ± 0.20	9.08 ± 0.10
IL-6	4 h	24.36 ± 1.85	13.62 ±0.34	20.66 ± 0.17	8.69 ± 0.10
IL-8	4 h	24.79 ± 0.82	10.06 ± 0.15	19.62 ± 0.06	9.10 ± 0.17
MMP-1	4 h	18.67 ± 0.12	15.75 ± 0.27	20.43 ± 0.16	14.28 ± 0.27
MMP-2	4 h	10.41 ± 0.65	10.50 ± 0.46	12.65 ± 0.22	12.60 ± 0.12
MMP-3	4 h	16.73 ± 0.20	11.38 ± 0.07	19.40 ± 0.14	10.41 ± 0.14
MMP-9	4 h	18.10 ± 0.19	16.03 ± 0.11	20.16 ± 0.25	19.16 ± 0.26
MMP-13	4 h	19.53 ± 0.65	14.09 ± 0.43	20.82 ± 0.21	19.09 ± 0.35
MIP-2	4 h	nd	nd	19.41 ± 0.19	9.54 ± 0.16
MIP-3	4 h	nd	nd	18.97 ± 0.13	9.68 ± 0.12
CCL5/RANTES	4 h	26.44 ± 0.01	16.50 ± 0.49	18.50 ± 0.18	12.37 ± 0.20

Table S2. *Cont.*

Gene	Time	SW1353 Unstimulated ΔCT ± st.dev. [1]	SW1353 IL-1b Stimulated ΔCT ± st.dev. [1]	NHAC-kn Unstimulated ΔCT ± st.dev. [1]	NHAC-kn IL-1b Stimulated ΔCT ± st.dev. [1]
CXCL10/IP-10	4 h	25.84 ± 0.28	14.35 ± 0.26	31.56 ± 2.32	20.64 ± 0.51
TIMP-1	4 h	10.33 ± 0.10	10.25 ± 0.31	8.39 ± 0.23	7.84 ± 0.10
COL2A1	4 h	20.33 ± 0.66	20.95 ± 0.82	22.34 ± 0.51	22.15 ± 0.27
Aggrecan	4 h	16.38 ± 0.12	15.49 ± 0.36	13.39 ± 0.18	13.59 ± 0.14
ADAMTS-4	4 h	23.31 ± 0.73	21.81 ± 0.36	21.98 ± 0.12	19.38 ± 0.45
LIF	4 h	18.63 ± 0.28	13.59 ± 0.32	16.77 ± 0.15	10.86 ± 0.16
ADAMTS-5	4 h	19.08 ± 0.87	19.50 ± 1.13	13.73 ± 0.20	13.38± 0.15

[1]: (threshold cycles for 18S rRNA [internal standard]—threshold cycles for genes of) ± st.dev.

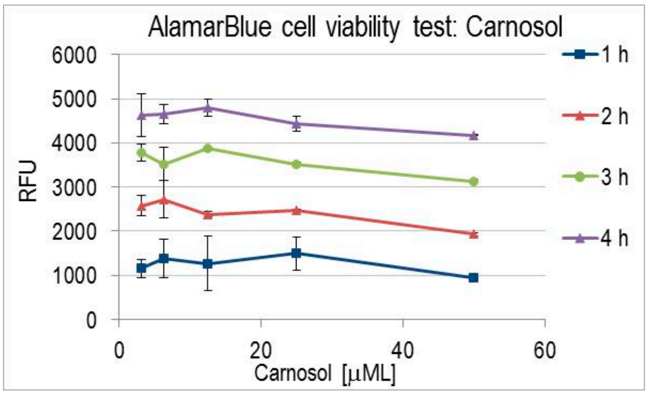

Figure S1. Freshly isolated PBLs were incubated with the indicated amounts of carnosol for 1-4 h. Using the Alamar Blue® cell viability test according to the manufacturers' instructions (ThermoFischer Scientific), the produced fluorescence was measured at the indicated time points. RFU, relative fluorescence units. In the absence of carnosol, cells produced similar RFU values as those observed in the presence of 3.125 to 25 µM carnosol.
